# Hypoxia-induced transcriptional differences in African and Asian versus European diabetic cybrids

**DOI:** 10.1038/s41598-023-30518-x

**Published:** 2023-03-07

**Authors:** Andrew H. Dolinko, Marilyn Chwa, Kevin Schneider, Mithalesh K. Singh, Shari Atilano, Jie Wu, M. Cristina Kenney

**Affiliations:** 1 Department of Pathology and Laboratory Medicine, University of California Irvine, Irvine, CA 92697 USA; 2 Department of Ophthalmology, Gavin Herbert Eye Institute, University of California Irvine, 843 Health Science Road, Hewitt Hall, Room 2028, Irvine, CA 92697-4390 USA; 3 Department of Biological Chemistry, University of California Irvine, Irvine, CA 92697 USA

**Keywords:** Biological techniques, Cell biology, Evolution, Molecular biology, Medical research

## Abstract

Diabetic retinopathy (DR) is the most common diabetic microvascular complication and cause of blindness in adults under the age of 65. Our results suggest that, when comparing transcriptomes of cultures grown in hypoxic conditions versus room-air, cybrids containing mitochondria from African and Asian diabetic subjects ([Afr + Asi]/DM) have some uniquely different transcriptome profiles compared to European/diabetic (Euro/DM) cybrids (e.g., fatty acid metabolism: EnrichR rank 10 in [Afr + Asi]/DM, rank 85 in Euro/DM; Endocytosis: rank 25 in [Afr + Asi]/DM, rank 5 in Euro/DM; Ubiquitin Mediated Proteolysis: rank 34 in [Afr + Asi]/DM, rank 7 in Euro/DM). As determined by both RNA-seq and qRT-PCR results, transcription of the gene encoding oleoyl-ACP hydrolase (*OLAH*) was significantly increased in [Afr + Asi]/DM cybrids compared to Euro/DM cybrids in hypoxic conditions. Additionally, our results show that in hypoxic conditions, Euro/DM cybrids and [Afr + Asi]/DM cybrids show similar decreases in ROS production. All cybrids showed decreased ZO1-minus protein levels, but their phagocytic functions were not significantly altered in hypoxic conditions. In conclusion, our findings suggest that the "molecular memory" imparted by [Afr + Asi]/DM mtDNA may act through one of the molecular pathways seen in transcriptome analysis, such as fatty acid metabolism, without significantly changing essential RPE functions.

## Introduction

Over the past 40 years, diabetes mellitus (DM) has become one of the most widespread diseases globally. Within the past decade, its prevalence increased from 366 million people in 2011 to 451 million in 2017, and it is predicted to further increase to 693 million people by 2045^1,2^. The characteristic hyperglycemia of this disease induces an inflammatory response that causes damage to blood vessels, resulting in the macrovascular (e.g., myocardial infarction) and microvascular complications, (e.g., nephropathy and peripheral neuropathy)^3^. Diabetic retinopathy (DR) is the most common diabetic microvascular complication and the most common cause of blindness in adults under the age of 65^4^. In DR, high glucose promotes non-enzymatic glycation of proteins, leading to oxidative stress and inflammation. Together, these elements damage the intraretinal vasculature, which supports the neurons of the inner retinal layers, and the choriocapillaris, which nourishes the photoreceptors in the outer retina^5^.

In healthy individuals, a blood-retina barrier prevents vascular contents from leaking into the retina. The endothelial cells that line the intraretinal vessels tightly bind to each other through tight junctions proteins; however, in DR and related conditions, such as diabetic macular edema (DME), this barrier becomes more permeable^6,7^. The choriocapillaris, which provides nutrients to the outer retina, is fenestrated and consequently more prone to leaking its contents. To prevent vascular leakage from entering the outer retina, retinal pigment epithelial (RPE) cells form a barrier that governs molecular transport between the outer retina and the choroid vessels. In diabetes, the RPE acquires damage that impairs its barrier functions, and these dysfunctions are implicated in the development of DR and DME^7,8^.

The RPE performs multiple functions in order to maintain a homeostatic balance with photoreceptors and preserve the architecture of the outer neural retina. Normally, photoreceptors shed their outer segments full of retinaldehyde proteins, and the RPE phagocytoses these segments to convert the *all-trans*-retinal into 11-*cis*-retinal that photoreceptors can use to form new rhodopsins^9,10^. In diabetes, RPE cells are suspected to have lower phagocytotic activity as shown in one study that reported decreased phagocytosis of rod outer segments in RPE cells exposed to high glucose in vitro^11^. In order to constantly recycle photoreceptor outer segments, RPE cells are highly metabolic, generating a substantial amount of reactive oxygen species (ROS) from mitochondrial oxidative phosphorylation (OXPHOS) in the process^12^. Diabetes leads to a decline in the ability of retinal cells to reduce ROS as levels of antioxidants, such as glutathione, are decreased^13^. Additionally, the RPE forms the outer portion of the blood-retina barrier through tight junctions proteins, such as zonula occludens-1 (ZO-1) and occludin^14,15^. In diabetes, these proteins are depleted, contributing to a decrease in barrier function^16–18^.

While many studies have examined the effect of diabetes-induced changes in mitochondrial function^19,20^ or the effect of mtDNA damage^21,22^ on cellular health, none have examined the role of mitochondrial haplogroups on RPE function in diabetes. The ancestral origin of individuals can be categorized into haplogroups, which are defined by an accumulation of specific single nucleotide polymorphisms (SNPs) within their mtDNA^23^. Previously, we demonstrated that mitochondria from diabetic patients of African or Asian ancestry confer resistance against hyperglycemic and hypoxic stressors in RPE cybrid cells^24^. In this investigation, we used our cytoplasmic hybrid (cybrid) RPE cells with mitochondria from diabetic (DM) or nondiabetic (non-DM) patients of African and Asian ([Afr + Asi]) or European (Euro) haplogroups to explore how mtDNA affects the transcriptomes and functions of RPE cells. Our findings from this study suggest that cybrids containing mitochondria from African and Asian diabetic [Afr + Asi]/DM] subjects have significantly different transcriptomes than Euro/DM cybrids cultured in hypoxic conditions. In addition, our results show that RPE cybrids with Euro/DM or [Afr + Asi]/DM exhibit similar changes in phagocytic function and tight junction protein expression when cultured in hypoxia. Overall, our findings suggest that the unique "molecular memory" imparted by [Afr + Asi]/DM mtDNA may occur through one of the molecular pathways observed in transcriptome analysis without changing the essential RPE functions.

## Materials and methods

### Human subjects

Research involving human subjects was approved by the Institutional Review Board of the University of California, Irvine (#2003-3131). All enrolled patients provided written, informed consent. Clinical investigations were performed based on the ethical principles of the Declaration of Helsinki^26^.

### Cybrid generation

Patient blood was collected in tubes containing 3.2% sodium citrate before being subjected to multiple centrifugations to isolate platelets. Platelet pellets were resuspended in Tris buffered saline (TBS). ARPE-19 cells, an RPE cell line (American Type Culture Collection (ATCC), Manassas, VA), were sequentially passaged in low-dose ethidium bromide to depleted cells of mtDNA (Rho*0*). Rho*0* ARPE-19 cells were cultured in standard culture media (10% FBS, 100 units/mL Penicillin, 100 mcg/mL Streptomycin, 25 μg/mL Fungizone (Amphotericin B: Omega Scientific, Torzana, CA), and 50 μg/mL Gentamycin in DMEM/F12) supplemented with uridine^25^. Then, platelets were fused with Rho*0* cells in polyethylene glycol-containing media in order to generate cybrids, as per a modified protocol by Chomyn^26^. Cybrids were cultured in standard culture media alone to select for RPE cells that successfully integrated mitochondria. The mtDNA incorporation was verified using a combination of polymerase chain reaction (PCR) and restriction enzyme digestion of these PCR products. In addition, mtDNA inclusion was verified through mtDNA sequencing to identify the mtDNA haplogroup for each cybrid^27^. The cohorts of Euro and [Afr + Asi] cybrids were generated from individuals of similar ages (*p* = 0.11), genders, and DM versus Non-DM status (Supplemental Table S1 and S2). In the present study, our cybrids were cultured in media containing 17.5 mM glucose.

### RNA-Seq analyses

*RNA Extraction*—Cybrids (Euro/Non-DM, n = 4; Euro/DM, n = 4; [Afr + Asi]/Non-DM, n = 4; and [Afr + Asi]/DM cybrids, n = 4) were cultured in 6-well plates at 500,000 cells/well in duplicate and incubated overnight at 37 °C with 5% CO_2_, then in 2 mL standard media at 37 °C with 5% CO_2_ in either room-air (~ 21% O_2_) or in a 2% O_2_ incubator (MCO-18 M O_2_/CO_2_ Incubator, Sanyo, Osaka, Japan) for 48 h. Duplicate cultures grown in identical conditions were combined and pelleted by centrifugation before isolating RNA using a PureLink RNA Mini Kit (Invitrogen, Waltham, MA) as per the manufacturer’s protocol. RNA was quantified on a NanoDrop 1000 spectrophotometer (Thermo Scientific, Waltham, MA).

*RNA Library Preparation and RNA-Seq*—RNA from each sample was analyzed for quality control through a combination of 2100 Bioanalyzer Nano RNA chip (Agilent Technologies, Santa Clara, CA) and NanoDrop ND-1000 (Thermo Scientific) absorbance ratios (260/280 nm and 260/230 nm). Library construction was performed as per the TruSeq Stranded mRNA protocol (Illumina, San Diego, CA). For each sample, 500 ng of total RNA was enriched for mRNA using oligo-dT magnetic beads and subsequently chemically-fragmented for three minutes. Then, first-strand synthesis was performed, using random primers and reverse transcriptase to make single-stranded cDNA. After second-strand synthesis, the resultant double-stranded cDNA was cleaned using AMPure XP beads (Beckman Coultier, Brea, CA). Cleaned cDNA was end-repaired, and then the 3’ ends were adenylated. Illumina barcoded adapters were ligated on the ends of the cDNA and the adapter-ligated fragments were enriched by nine cycles of PCR. The resulting libraries were validated by qPCR and sized by the Agilent Bioanalyzer DNA high sensitivity chip. The concentrations for the libraries were normalized before multiplexing the libraries together and then sequenced using single end 100 cycles chemistry on a HiSeq 4000 sequencer (Illumina).

*RNA-Seq Output Analysis*—After acquiring the reads, the data were analyzed for quality control using *FastQC*. Reads were then quality and adaptor trimmed using *Trimmomatic*^28,29^. Trimmed reads were then aligned to the human hg19 reference genome using a splice aware short read sequence alignment tool, *TopHat2*^30^. Gene expression was quantified by raw read counts using *featureCounts* and Reads-Per-Kilobase-per-Million mapped reads (RPKM) using *Cufflinks*^31,32^.

Quantified data were then placed into 8 groups based on whether the cybrid from which the RNA originated had Euro or [Afr + Asi] mtDNA, was from a DM or Non-DM patient, or was grown in room-air or hypoxia. Differential gene expression analysis between selected groups was performed using an *R*-based tool, *DESeq2*^33^. (Negative Binomial model, wald test) with a FDR adjusted p value threshold 0.1.

Differentially-expressed gene lists from comparisons of DM cybrids in room-air with their respective hypoxic samples ([Afr + Asi/DM/Room-Air] versus [Afr + Asi/DM/Hypoxia]) and ([Euro/DM/Room-Air] versus [Euro/DM/Hypoxia]) were analyzed for pathway enrichment terms using EnrichR^34–36^. The top 10 differentially-expressed pathways for each comparison were presented, using the Kyoto Encyclopedia of Genes and Genomes 2016 (KEGG 2016) classification where unique pathways in the top 10 lists were highlighted.

### ROS assay

ROS levels were measured in our cybrids (n = 4 for all groups) using a variation of the methods we have previously described^37^. Experiments were performed in sextuplicate. Briefly, cybrids were plated in 100 μL of standard culture media at a density of 10,000 cells/well in 2 separate 96-well, black well, clear-bottom plates, incubated overnight at 37 °C with 5% CO_2_, and subsequently incubated 48 h at 37 °C with 5% CO_2_ in either room-air or 2% O_2_. After incubation, media were replaced with 100 μL of 10 μM dichlorodihydrofluorescein diacetate (H_2_DCFDA; Invitrogen) in Dulbecco’s PBS (DPBS; Gibco, ThermoFisher Scientific, Waltham, MA), and plates were incubated at 37 °C without additional CO_2_ for 30 min. Subsequently, H_2_DCFDA solution was removed and replaced with 100 μL DPBS. Fluorescence was measured using a SpectraMax Gemini XPS fluorescent plate reader (Molecular Devices, San Jose, CA) with excitation at 492 nm and emission at 520 nm. For each cybrid, values were normalized to the average fluorescence of samples cultured in room-air.

### Phagocytosis assay

Phagocytosis was measured in our cybrids (n = 4 Euro/Non-DM, n = 5 Euro/DM, n = 3 [Afr + Asi]/Non-DM, n = 3 [Afr + Asi]/DM) using a variation of the methods described by Vo et al.^38^ All experiments were performed in triplicate.

*Culture of Cybrids With Fluorescent Beads*—For each cybrid, cells were seeded at a density of 500,000 cells/well in 2 mL of standard culture media in 2 separate 6-well plates and then incubated overnight at 37 °C with 5% CO_2_. Media were replaced with 2 mL of a solution of 1 μm, fluorescently-tagged latex microbeads (Fluoresbrite® YG Microspheres 1.00 μm; Polysciences, Inc., Warrington, PA) diluted 1:3,000 in standard culture media. For each cybrid, 1 plate was incubated 48 h at 37 °C with 5% CO_2_ in 2% O_2_ while the other plate was incubated for 48 h at 37 °C with 5% CO_2_ in room-air.

After incubation, wells were briefly washed 3 times with 2 mL PBS-EDTA per wash and trypsinized. For each well, 2 mL of standard medium was added before pipetting each content into a separate 15 mL conical tube. Then, 1.5 mL of standard media was added to each well and then added to its respective 15 mL conical tube. Cell suspensions were then titrated with a 5 mL pipette to separate into single cells, strained through separate 35 μm nylon filters into separate 5 mL test tubes (Corning™ Falcon™ Test Tube with Cell Strainer Snap Cap; Corning Inc., Corning, NY), and centrifuged for 5 min at 1000 RPM. For each tube, media were carefully removed, and the pellet was resuspended in 50 μL of PBS-EDTA.

*Flow Cytometry Analysis of Phagocytosis*—Each sample was loaded into an ImageSteamX Mark II Imaging Flow Cytometer (Luminex Corp., Austin, TX), excited using a 488 nm laser, and imaged at 40 × magnification. 5,000 images were collected for each sample.

Imageset data were then analyzed using IDEAS software (Luminex Corp.) Briefly, images were first gated by object diameter to isolate images with single cells. Then, this subset was gated by fluorescence to determine the images containing microspheres. Finally, this smaller subset was gated to identify images where microspheres had been internalized. To do this, the software generates a “mask” of the cell area, “erodes” this area by a few pixels from the outer edge, and then determines if a fluorescent signal is located within the eroded mask. An internalization ratio was then calculated as follows: [Single Cells that Internalized Beads] / [Total Single Cells]. For each cybrid and condition, this ratio was then normalized to the ratio of its age-matched and haplogroup-matched, Non-DM cybrid cultured in room-air.

### Western blot of tight-junctions proteins

*Protein Extraction from Cybrid Cultures*—For each cybrid, cells were seeded at a density of 500,000 cells/well in 2 mL of standard culture media in all wells of 2 separate 6-well plates and then incubated at 37 °C with 5% CO_2_ for 19 days. Then, for each cybrid, 1 plate was incubated 48 h at 37 °C with 5% CO_2_ in 2% O_2_ while the other plate was incubated under same conditions in room-air. Each well was lysed in 250 µL of a radioimmuniproecipitation assay (RIPA) buffer-based solution (RIPA Lysis Buffer (MilliporeSigma, Burlington, MA), PhosphataseArrest™ I (Gbiosciences, St. Louis, MO), and Protease Inhibitor Cocktail (MilliporeSigma). For each plate, lysates from wells in the same column were combined and placed in a 1.5 mL microcentrifuge tube. Lysates were centrifuged for 15 min at 14,000 × g at 4 °C and supernatants were collected. Clarified lysate protein concentrations were measured using the Pierce™ BCA Protein Assay kit (Thermo Scientific). Experiments were performed in triplicate.

*Immunoblotting for Tight Junctions Proteins*—For each sample, 75 μg of protein was denatured in protein loading buffer (lauryl dodecyl sulfate (Bolt™ LDS Sample Buffer; Life Technologies, ThermoFisher Scientific); dithiothreitol (Bolt™ Sample Reducing Agent; Life Technologies) at 95 °C for 5 min. Twenty μg of protein in loading buffer of each sample was loaded into the wells of 4–12% Bolt™ Mini Gels (Life Technologies) with protein ladder (Precision Plus Protein™ Dual Color Standards; BioRad, Hercules, CA) and subjected to electrophoresis for 1–2 h at 100 V. Proteins were then transferred from the gels onto PVDF membranes (ImmunBlot®; BioRad) using wet transfer for 1 h at 20 V. Membranes were blocked in 5% milk in 1 × TBST (Tris Base (Trizma®; MilliporeSigma), 0.1% Tween-20 (Fisher Scientific, ThermoFisher Scientific) ) at room temperature for 1 h. The membrane was then cut horizontally in parts at 75 kDa ladder band with one part containing proteins less than 75 kDa and the other part containing proteins larger than 75 kDa. The membrane carrying less than or equal to 75 kDa was incubated overnight at 4 °C in 5% milk in 1 × TBST with β-actin and Occludin antibodies, while the other part of the membrane was incubated with Z1 antibody (Supplemental Table S3).

Membranes were washed in 1 × TBST and incubated with secondary antibody (Supplemental Table S3 in 5% milk in 1 × TBST for 1 h at room temperature. Membranes were washed again in 1 × TBST. Protein bands were detected using SuperSignal™ West Femto Maximum Sensitivity Substrate (Thermo Scientific) as per manufacturer’s instructions. The two parts of the same membrane were wrapped in plastic wrap and placed in gel doc imaging. We took a bright field image prior turning on the ECL camera to show that the two parts of the membrane were cut from the same membrane but stained with different antibodies. β-actin was used as a housekeeping protein control. Chemiluminescent images were captured using a ChemiDoc MP imager (BioRad) and quantified using ImageJ (National Institutes of Health, Bethesda, MD).

### Statistical analysis

Unless otherwise indicated, data were compared using Unpaired Student’s *t*-test or One-Way ANOVA with Bonferonni’s Post-Test (GraphPad Prism, version 5.0, GraphPad Software, CA). Results were adjusted using a Bonferonni correction where appropriate. Results with *p* ≤ 0.05 were considered statistically significant.

### Ethics approval

This study was performed in line with the principles of the Declaration of Helsinki. Approval was granted by the University of California, Irvine’s Institutional Review Board (UCI IRB#2003-3131).

### Consent to participate

Informed consent was obtained from all individual participants included in the study.

## Results

### African + Asian and European cybrids show unique differences in transcriptomes between hypoxic and room-air cultures

The molecular mechanisms underlying our previous observations were characterized by performing high-throughput RNA-seq on our cybrid population in room-air and hypoxic conditions. The transcriptomes of the DM cybrids cultured in room-air and hypoxia were compared as follows: [Afr + Asi]/DM/Room-Air versus [Afr + Asi]/DM/Hypoxia (Table 1) and Euro/DM/Room-Air versus Euro/DM/Hypoxia (Table 2). Genes that were significantly expressed between the two conditions were then analyzed using the EnrichR online software to determine the top-ranked, differentially-expressed pathways, as categorized by the KEGG 2016 enrichment terms. For both the Euro and [Afr + Asi] comparisons, differential gene expression of certain pathways was highly-ranked, such as those involving protein processing in the endoplasmic reticulum (3rd for [Afr + Asi], 2nd for Euro), metabolic pathways (2nd for [Afr + Asi], 3rd for Euro), and carbon metabolism (4th in both lists) (Tables 1 and 2). However, other pathways were more highly-ranked for the Euro/DM cybrids than the [Afr + Asi]/DM cybrids. In particular, more genes in pathways associated with endocytosis (25th for [Afr + Asi]/DM, 5th for Euro/DM) and degradation of ubiquitylated proteins (34th for [Afr + Asi]/DM, 7th for Euro/DM) were differentially-expressed in the Euro/DM cybrids comparison than that of the [Afr + Asi]/DM cybrids. In addition, more genes in pathways associated with fatty acid metabolism were differentially-expressed in the comparison of [Afr + Asi]/DM cybrid transcriptomes than in the comparison of Euro/DM transcriptomes (10th for [Afr + Asi], 85th for Euro). These findings indicate that the Euro/DM mitochondria differentially modulate the nuclear gene expression compared to the [Afr + Asi]/DM mitochondria because all of the cybrid cell lines possess identical nuclei but differ only in the mitochondria from different haplogroup populations.

**Table 1:** EnrichR Pathway analysis of differentially-expressed genes between Afr + Asi/DM cybrids cultured in room air versus 2% oxygen.

African and Asian [Afr + Asi] DM, hypoxia versus room air					
Rank	Term	Overlap	Adjusted *p*-value	Z-score	Combined score
1	Ribosome_Homo sapiens_hsa03010	83/137	3.36816E−21	− 1.74614	92.23118
2	Metabolic pathways_Homo sapiens_hsa01100	378/1239	1.30460E−13	− 1.98676	68.85048
3	Protein processing in endoplasmic reticulum_Homo sapiens_hsa04141	70/169	1.77845E−07	− 1.76655	35.54995
4	Carbon metabolism_Homo sapiens_hsa01200	49/113	6.29999E−06	− 1.63462	26.59338
5	Lysosome_Homo sapiens_hsa04142	50/123	4.15835E−05	− 1.67413	23.70320
6	Valine, leucine and isoleucine degradation_Homo sapiens_hsa00280	25/48	1.09485E−04	− 1.77390	23.07510
7	TNF signaling pathway_Homo sapiens_hsa04668	43/110	5.61712E−04	− 1.75310	19.66767
8	Neurotrophin signaling pathway_Homo sapiens_hsa04722	45/120	9.57908E−04	− 1.67261	17.45152
9	Inflammatory mediator regulation of TRP channels_Homo sapiens_hsa04750	38/98	1.15460E−03	− 1.66358	16.43489
10	Fatty acid metabolism_Homo sapiens_hsa01212	23/48	9.70756E−04	− 1.45318	14.98963
11	HIF-1 signaling pathway_Homo sapiens_hsa04066	39/103	1.39184E−03	− 1.43983	13.65642
12	Insulin resistance_Homo sapiens_hsa04931	40/109	2.37148E−03	− 1.43738	12.78004
18	AGE-RAGE signaling pathway in diabetic complications_Homo sapiens_hsa04933	36/101	7.02269E−03	− 1.43281	10.75077
25	Endocytosis_Homo sapiens_hsa04144	75/259	1.54455E−02	− 1.17626	7.47406
34	Ubiquitin mediated proteolysis_Homo sapiens_hsa04120	43/137	2.17697E−02	− 0.66996	3.84699

The top 10 pathways, along with others that are much more differently ranked between the Euro/DM and [Afr + Asi]/DM comparisons are listed.

**Table 2:** EnrichR pathway analysis of differentially-expressed genes between Euro/DM cybrids cultured in room air versus 2% oxygen.

European (Euro) DM, Hypoxia versus room air					
Rank	Term	Overlap	Adjusted *p*-value	Z-score	Combined score
1	Ribosome_Homo sapiens_hsa03010	85/137	1.47075E−21	− 1.74614	93.67801
2	Protein processing in endoplasmic reticulum_Homo sapiens_hsa04141	71/169	5.56805E−07	− 1.79043	34.71290
3	Metabolic pathways_Homo sapiens_hsa01100	352/1239	2.40768E−06	− 1.96192	34.36975
4	Carbon metabolism_Homo sapiens_hsa01200	46/113	4.07692E−04	− 1.63462	19.77707
5	Endocytosis_Homo sapiens_hsa04144	84/259	2.87911E−03	− 1.83881	17.86864
6	AGE-RAGE signaling pathway in diabetic complications_Homo sapiens_hsa04933	39/101	3.61021E−03	− 1.83334	16.69610
7	Ubiquitin mediated proteolysis_Homo sapiens_hsa04120	51/137	2.00388E−03	− 1.54990	15.93823
8	HIF-1 signaling pathway_Homo sapiens_hsa04066	40/103	2.87911E−03	− 1.65506	15.64200
9	Lysosome_Homo sapiens_hsa04142	46/123	2.87911E−03	− 1.62388	15.51125
10	Insulin resistance_Homo sapiens_hsa04931	41/109	3.93832E−03	− 1.59642	14.07932
85	Fatty acid metabolism_Homo sapiens_hsa01212	18/48	6.91730E−02	0.12171	− 0.55465

The top 10 pathways, along with others that are much more differently ranked between the Euro/DM and [Afr + Asi]/DM comparisons are listed.

**Figure 1:**
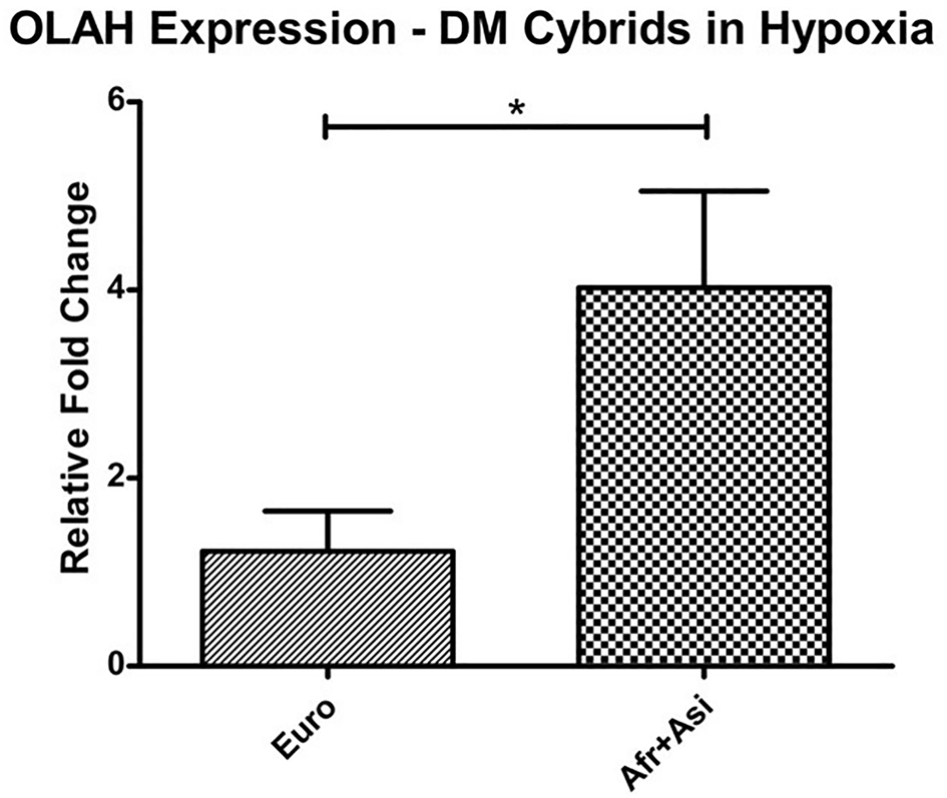
[Afr + Asi]/DM cybrids express more OLAH transcript in hypoxia than similarly-treated Euro/DM cybrids. RNA from DM cybrids cultured 48 h in 2% O_2_ was analyzed by qRT-PCR to measure transcript levels of the Oleoyl-ACP Hydrolase (*OLAH*) gene and compare expression between Euro/DM and [Afr + Asi]/DM cybrids. The data were normalized to average OLAH expression in Euro/DM cybrids (Fold-Change = 1).

In addition, we performed a pairwise comparison of transcriptome data between Euro/DM cybrids and [Afr + Asi]/DM cybrids cultured in hypoxia. The analysis revealed that the transcript for oleoyl-ACP hydrolase (OLAH), an enzyme involved in medium fatty acid chain synthesis, was significantly more enriched in [Afr + Asi]/DM cybrids than Euro/DM cybrids (Table 3). qRT-PCR for *OLAH* in our DM/Hypoxia samples revealed that *OLAH* was approximately fourfold higher in [Afr + Asi] samples than Euro samples (*p* = 0.045) (Fig. 1). Moreover, the mitochondrial DNA (mtDNA) copy numbers of Non-DM and DM cybrids were not significantly different for both groups of cybrids (Euro/DM: 0.84 ± 0.19—fold of Euro/Non-DM, *p* = 0.38; Afr + Asi/DM: 0.98 ± 0.13—fold of Afr + Asi/DM, *p* = 0.89) (Supplemental Figure S1).Overall, these results suggest that genes in the pathways of endocytosis, ubiquitylated protein degradation, and fatty acid metabolism (such as *OLAH*) are differentially-expressed when different mtDNA haplogroup profiles are present.

**Table 3:** The *OLAH* transcript is more enriched in [Afr + Asi]/DM cybrids cultured in hypoxia than similarly-treated Euro/DM cybrids.

DM/Hypoxia, Euro versus [Afr + Asi]		
Gene	Log(2) of fold-change	Adjusted *p* value
OLAH	− 2.459220449	0.009283141

Pairwise analysis of transcripts from Euro/DM/Hypoxia and [Afr + Asi]/DM/Hypoxia cultures. Results were normalized to the [Afr + Asi]/DM/Hypoxia transcripts (Log(2) of Fold Change for Non-Euro = 0).

### ROS levels decrease in hypoxia for European and African + Asian cybrids

**Figure 2:**
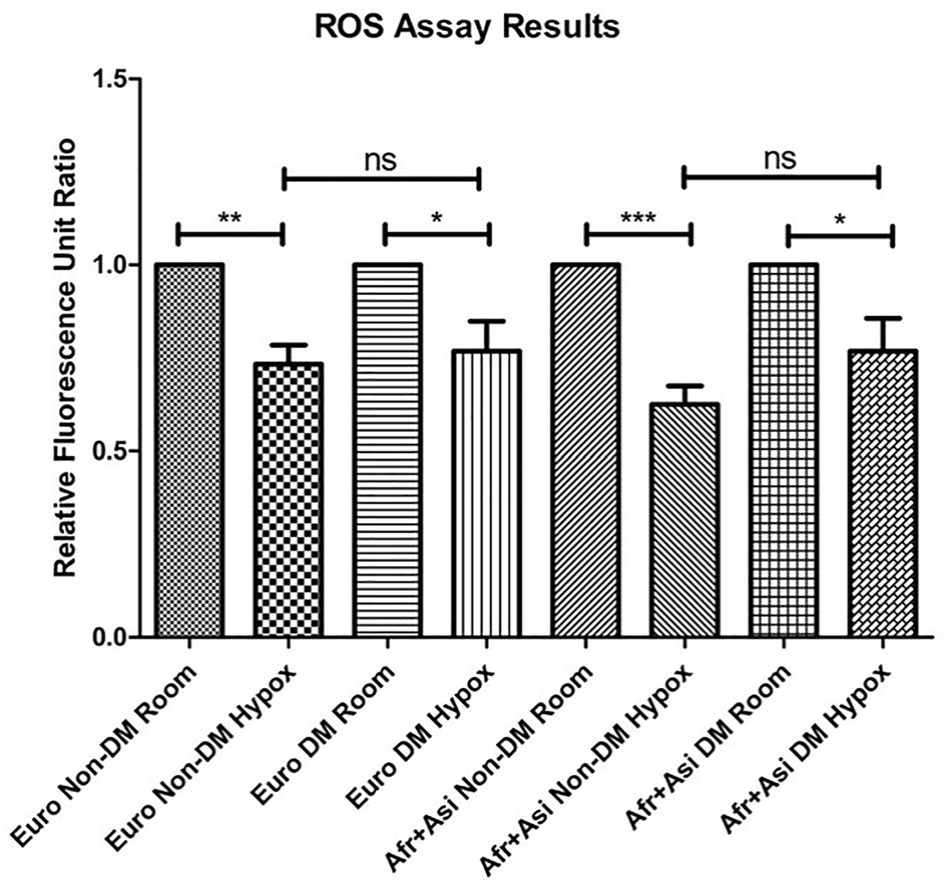
ROS levels decrease in hypoxia for European and [Afr + Asi], DM and Non-DM cybrids. ROS levels in cybrids cultured 48 h ± 2% O_2_ was measured using an H_2_DCFDA-based assay. For each group of cybrids, data were normalized to relative fluorescence of cells cultured in room air.

To determine if mtDNA haplogroup and diabetes status affect the cellular functions of RPE cells, we performed an assay to detect ROS levels in our cybrids (Fig. 2). In hypoxic conditions, ROS levels significantly decreased for all groups as compared to cybrid cultures grown in room-air (Euro/Non-DM: 0.73 ± 0.05-fold of ROS levels in room-air cultures, *p* = 0.0020; Euro/DM: 0.77 ± 0.08-fold, *p* = 0.029; [Afr + Asi]/Non-DM: 0.62 ± 0.05-fold, *p* = 0.0003; [Afr + Asi]/DM: 0.77 ± 0.09,-fold *p* = 0.039) (Fig. 2). For both Euro and [Afr + Asi] haplogroup cohorts, the relative ROS levels were not significantly different between DM and Non-DM cybrids cultured in hypoxia (Euro/Non-DM/Hypoxia vs. Euro/DM/Hypoxia, *p* > 0.05; [Afr + Asi]/Non-DM/Hypoxia vs. [Afr + Asi]/DM/Hypoxia, *p* > 0.05). Altogether, these findings suggest that ROS levels similarly decrease in RPE cells in hypoxia, regardless of mtDNA haplogroup and diabetes history.

### European and African + Asian cybrids retain phagocytic function in hypoxia

**Figure 3:**
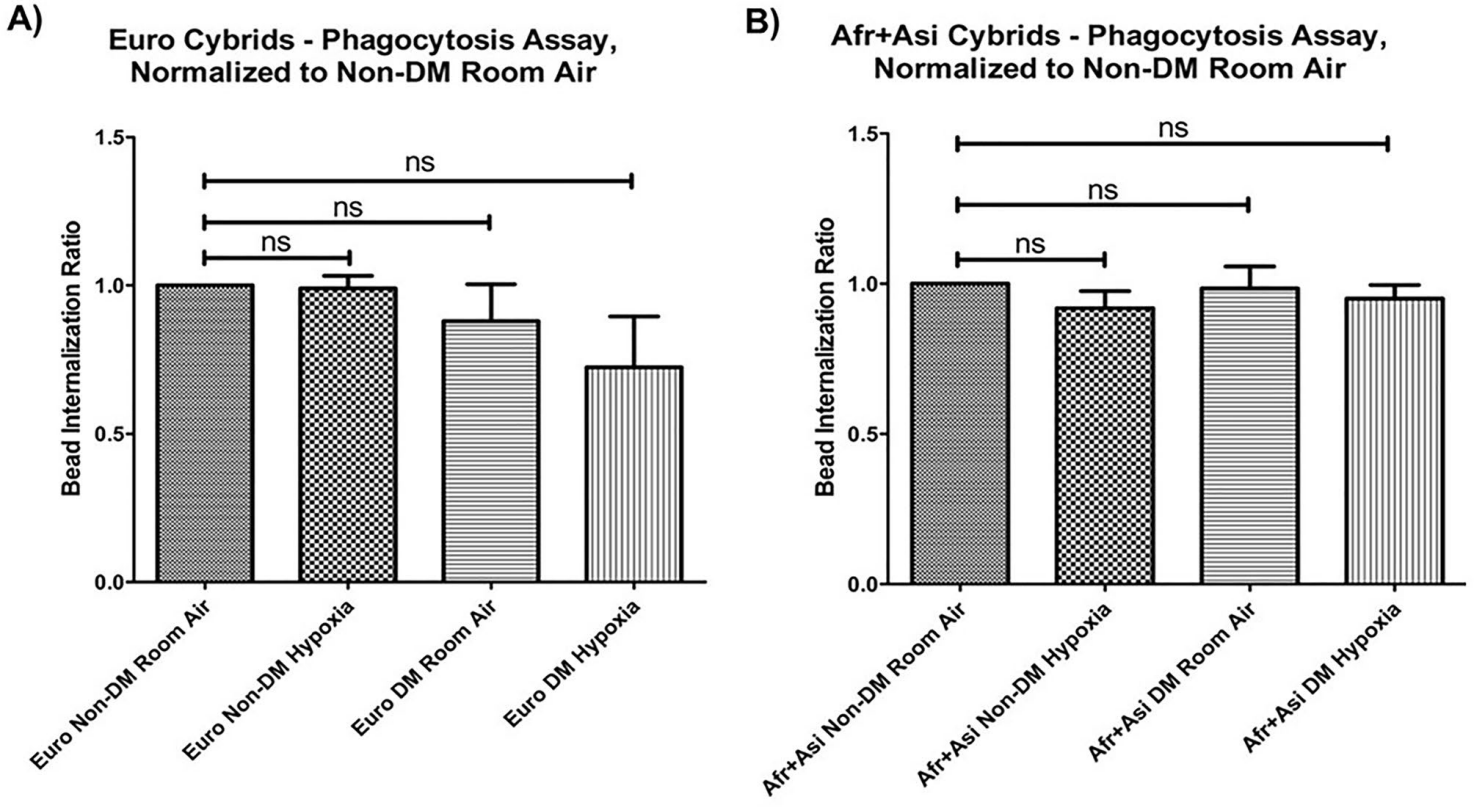
Phagocytic function is retained in hypoxia for European and African + Asian, DM and Non-DM cybrids. Phagocytosis in our cybrids cultured 48 h ± 2% O_2_ was measured using a fluorescent bead internalization-based assay for (**A**) European cybrids and (**B**) [Afr + Asi] cybrids. For both groups of cybrids, data were normalized to bead internalization for Non-DM cells cultured in room air. Afr + Asi = African + Asian; Euro = European; DM = Diabetic.

To determine if mtDNA haplogroup differentially influences phagocytic activity, cybrids were cultured in the presence of 1 μm fluorescent beads for 48 h in either room-air or 2% O_2_, and bead internalization was measured using flow cytometry. In room-air, the Euro/Non-DM cybrids and Euro/DM cybrids showed similar phagocytic activity of internalized beads phagocytic activity (0.88 ± 0.13-fold, *p* = 0.42). In addition, phagocytic activity showed a decreased, though not statistically significant, trend in phagocytosis when Euro/Non-DM or Euro/DM cybrids were cultured in hypoxia (Euro/Non-DM/Hypoxia: 0.98 ± 0.04-fold, *p* = 0.81; Euro/DM/Hypoxia: 0.72 ± 0.17-fold, *p* = 0.20) (Fig. 3A). Phagocytic activities of [Afr + Asi] cybrids, both diabetic and non-diabetic, were mostly preserved in hypoxic conditions ([Afr + Asi]/Non-DM/Hypoxia: 0.92 ± 0.06-fold of internalized beads in [Afr + Asi]/Non-DM/Room-Air cultures, *p* = 0.22; [Afr + Asi]/DM/Hypoxia: 0.95 ± 0.05-fold, *p* = 0.33). The phagocytic activity in [Afr + Asi]/DM cybrids cultured in room-air was comparable to that in similarly-treated [Afr + Asi]/Non-DM cybrids (0.98 ± 0.07-fold, *p* = 0.84) (Fig. 3B). In our RNA-Seq data, transcripts of genes involved in phagocytosis, such as clathrin (*CTLA*), *MERTK*, and *RAC1*, showed similar changes between room-air and hypoxic cultures for all cybrids (Supplemental Table 4). Overall, these data suggest that phagocytosis is not significantly altered in hypoxia, regardless of mitochondrial diabetic status and mtDNA haplogroup.

### European and African + Asian cybrids show decreases in ZO-1α-minus protein in hypoxia

**Figure 4:**
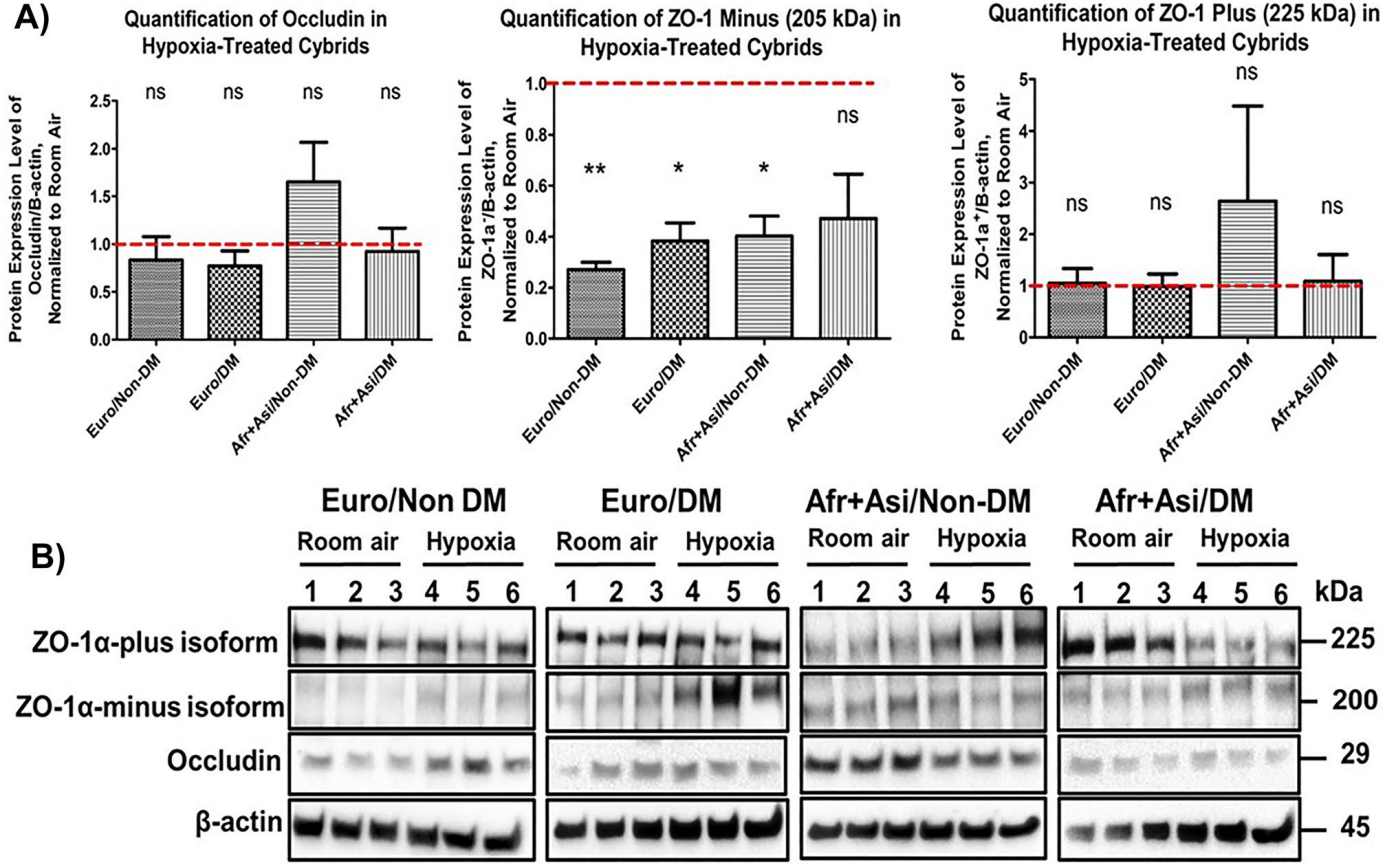
Tight junction protein levels are similarly changed in hypoxia for European and [Afr + Asi], DM and Non-DM cybrids. Tight junction proteins in our cybrids cultured 48 h ± 2% O_2_ was measured using a western blot assay for (**A**) occludin, (**B**) the 205 kDa ZO-1α-minus isoform, and (**C**) the 225 kDa ZO-1α-plus isoform. For both groups of cybrids, data were normalized to protein levels in cybrids cultured in room air (red dotted line, value = 1). Afr + Asi = African + Asian; Euro = European; DM = Diabetic.

Since the RPE forms part of the blood-retina barrier through tight junctions, we investigated whether mtDNA background and diabetes history affect levels of tight junction proteins, such as ZO-1 and occludin (Fig. 4A,D). For all groups, cybrids cultured in 2% O_2_ showed levels of occludin that were not significantly different from those in cybrids cultured in room-air (Euro/Non-DM: 0.84 ± 0.25-fold of protein levels in room-air cultures, *p* ≈ 1; Euro/DM: 0.77 ± 0.16-fold, *p* = 0.58; [Afr + Asi]/Non-DM: 1.65 ± 0.42-fold, *p* = 0.57; [Afr + Asi]/DM: 0.92 ± 0.25-fold, *p* ≈ 1). In contrast, *OCLN* transcripts were lower in most hypoxia-treated cultures than in room-air cultures in the RNA-Seq data, suggesting that future production of occludin protein would be similarly decreased among all cybrids cultured in hypoxia (Supplemental Table 5). Using western blot analyses, the ZO-1 protein levels were examined (Fig. 4B,D). The cybrid cultures expressed two different isoforms, ZO-1α-plus and ZO-1α-minus, which differ by a span of 80 amino acids. While ZO-1α-plus is expressed on the surface of many epithelial cell types, the ZO-1α-minus isoform is only expressed on endothelial cells and specialized epithelial cells, including Sertoli, renal glomerular, and RPE cells^39,40^. For all groups, cybrids cultured in 2% O_2_ exhibited levels of ZO-1α-plus that were not significantly different to those in cells cultured in room-air (represented by red dotted line, value = 1) (Euro/Non-DM: 1.04 ± 0.29-fold, *p* ≈ 1; Euro/DM: 0.98 ± 0.25-fold, *p* ≈ 1; [Afr + Asi]/Non-DM: 2.64 ± 1.84-fold, *p* ≈ 1; [Afr + Asi]/DM: 1.08 ± 0.52,-fold *p* ≈ 1) (Fig. 4C). On the other hand, ZO-1α-minus levels were significantly lower in hypoxia-treated cultures than those of cells cultured in room-air for Euro/Non-DM (0.27 ± 0.03-fold, *p* < 0.0003), Euro/DM (Euro/DM: 0.38 ± 0.07-fold, *p* = 0.0003), and [Afr + Asi]/Non-DM cybrids (0.40 ± 0.08-fold, *p* = 0.0048). ZO-1α-minus levels showed a decreased trend in [Afr + Asi]/DM cybrids, though this difference was not statistically significant (0.47 ± 0.17-fold, *p* = 0.11) (Fig. 4B,D). This finding is supported by the RNA-seq results showing that *TJP1* (ZO-1 gene) transcripts were lower in all hypoxia-treated cultures than in room-air cultures, based on the RNA-Seq results (Supplemental Table 4). Overall, these data suggest that mtDNA haplogroup and diabetes status do not significantly alter levels of tight junction proteins in hypoxia.

## Discussion

In diseases such as DR and age-related macular degeneration (AMD), hypoxia induces changes within the RPE. Previous studies have shown that RPE cells exposed to hypoxia have decreased ATP, lower cytochrome oxidase activity, and increased levels of cytokines compared to those exposed to room-air^41–43^. In this study, our RNA-seq data showed that when we compared transcriptomes of DM cybrids cultured in hypoxia to those in room-air, certain gene pathways were more highly enriched for African + Asian [Afr + Asi] cybrids than European (Euro) cybrids, and vice-versa. In particular, we found that Euro cybrids in hypoxia versus room-air had more differentially-expressed genes associated with endocytosis and ubiquitin-mediated proteolysis than [Afr + Asi] cybrids. This suggests that the initial response of Euro cybrids to hypoxia uniquely involves modification of these pathways, and that further experiments are needed to clarify how the mtDNA modifies these pathways and which factors are specifically targeted. Similarly, we found that [Afr + Asi] cybrids in hypoxia versus room-air had more differentially-expressed genes associated with fatty acid metabolism than Euro cybrids. Since we found that [Afr + Asi]/DM cybrids showed increased resistance to hypoxic and hyperglycemic stresses, one may speculate this pathway contributes to the protection imparted by [Afr + Asi]/DM mtDNA.

Furthermore, *OLAH* expression was significantly elevated in [Afr + Asi]/DM cybrids compared to Euro/DM cybrids. *OLAH* encodes for Oleoyl-ACP Hydrolase, an enzyme involved in medium fatty-acid chain synthesis. Originally called thioesterase II, OLAH was observed to be specifically expressed in normal and tumor-derived breast epithelial tissue, with elevated serum levels in rat models of breast cancer^44,45^. However, expression of *OLAH* can be induced in other cell types in disease conditions. For example, monocytes from patients affected by rheumatoid arthritis expressed significantly higher levels of *OLAH* than monocytes from patients with osteoarthritis^46^. In addition, in a study of children infected by an influenza virus, *OLAH* was significantly enriched in patients who exhibited neurologic symptoms, such as seizures or loss of consciousness, as well as in patients with pneumonia compared to patients without any of these symptoms^47^.

This is particularly interesting as the pathology of retinal diseases, such as accumulation of lipid-rich drusen deposits in the Bruch’s membrane in AMD, is associated with altered fat metabolism of RPE cells^48^. In particular, the Friedlander group generated mouse models of hypoxia that lack Von-Hippel Lindau protein, which inhibits hypoxia-inducible factors (Hifs), and found these mice had thickened choroid with dilated blood vessels. However, double knockouts of *Vhl* and *Hif2a* did not show changes in choroid thickness, suggesting that the process is driven by *Hif2a*. Similarly, microarray analysis revealed that the hypoxia mouse model significantly downregulated levels of thioesterases, such as *Acot7* and *Acot8*, compared to uninduced littermates, while this was not observed in the *Vhl* / *Hif2a* double knockouts^49^. Their study suggested that enzyme activity similar to that of OLAH is decreased in hypoxic RPE. Since we found that [Afr + Asi]/DM cybrids thrive in hypoxic conditions while this was not observed in the Euro/DM cybrids, one may speculate that the increased *OLAH* expression in [Afr + Asi]/DM cybrids allows them to use fatty acids for metabolism and avoid the accumulation of lipid deposits in RPE correlated with disease.

Since the RPE expend large amounts of energy to maintain the outer retinal microenvironment^50^, it generates high levels of ROS that must be managed. Previous work has shown that elevated ROS develop in RPE models of AMD and DR, particularly in response to hyperglycemia^51^. In our study, we discovered that hypoxia induced significant decreases in ROS for all cybrids tested. In addition, the decrease in ROS in hypoxic conditions was similar for both DM and non-DM cybrids, for both Euro and [Afr + Asi] cybrids. These data are consistent with our previous work showing that Euro cybrids and [Afr + Asi] cybrids have similar mitochondrial bioenergetics profiles^24^. One reason why hypoxic conditions induce decreased ROS in our cybrids may be a shared adaptive mechanism. Work by the Semenza group showed that mouse epithelial fibroblast cells decrease ROS when cultured in 1% oxygen due to a Hif-1α-induced mitochondrial autophagy pathway^52^. Overall, our data suggest that mtDNA haplogroup and diabetic status do not significantly affect ROS levels in response to hypoxia.

Phagocytosis of sloughed outer segments of photoreceptors is an important function of RPE cells. Previous work has shown that macrophages from diabetic mouse models have impaired phagocytosis that was correlated with increased glycated protein levels^53^. Additionally, mouse RPE cells exposed to high glucose levels in vitro exhibit impaired phagocytic function^18^. However, our cybrids did not show significant decreases in phagocytic function in cells exposed to hypoxia compared to untreated cultures. Moreover, when the transcript levels of key phagocytosis pathway factors were examined, we found they changed similarly between hypoxic and room-air conditions for all cybrids tested. This finding is similar to results we obtained when examining phagocytic activity in cybrids with mtDNA from patients with AMD or unaffected patients. When cybrids from either group were treated with the anti-VEGF compounds bevacizumab (Avastin®), ranibizumab (Lucentis®), or aflibercept (Eylea®), phagocytic function was decreased, with similar fold-changes in function between AMD and normal cybrids^38^. However, our data showed that the differential expression of endocytosis genes in hypoxic cybrids was more highly ranked for Euro/DM cybrids than for [Afr + Asi]/DM cybrids. Since Euro/DM cultures demonstrated a decreased trend in phagocytosis (though not significant), we may speculate that the Euro/DM cybrids increase transcription of phagocytic factors to recover from an initial decrease in phagocytic function. Overall, these data suggest that the metabolic memory imparted by mtDNA does not significantly impact phagocytic function in the presence or absence of hypoxic stress. However, given the decreased trend in phagocytosis in Euro/DM cybrids, these experiments warrant further investigation.

Another RPE function is to form the outer portion of the blood-retina barrier to prevent leakage of fluid from the choroid vasculature into the neural retina. Experiments in diabetic animal models and human RPE cells stressed with diabetic conditions show decreased levels and disorganization of tight junctions proteins, such as occludin and ZO-1, decrease in amount and become disorganized, resulting in increased retinal permeability^16,54^. Consistent with these findings, we observed significant decreases in the ZO-1α-minus protein isoform for Euro/Non-DM, Euro/DM, and [Afr + Asi]/Non-DM cybrids cultured in hypoxia compared to protein levels of cells in room-air; [Afr + Asi]/DM cybrids showed a decreased trend in ZO-1α-minus protein levels. We also observed that non-DMs responded more to hypoxia in ZO-1 minus hypoxia-treated cybrids than DM cybrids. It is well known that the retinal pigment epithelium secretes more VEGF in a hypoxic environment. ^49^ Other research reveals that the expression of ZO-1 alpha plus and ZO-1 alpha minus is downregulated in vascular endothelial cells but upregulated in retinal pigmented epithelial cells in response to VEGF treatment. ^40^ These findings imply that hypoxia and VEGF treatment would have the same effect on retinal pigment epithelium cells. In our study, we used Rho*0* cells from ARPE19 to create non-DM/DM cybrids. As a result, regardless of DM/non-DM status, there is an increase in ZO-1 alpha minus expression in response to hypoxia because it carries the common ARPE19 nucleus. Previous work described that while tight junctions containing ZO-1α-plus exhibit a more constant resistance, tight junctions containing ZO-1α-minus may fluctuate in their resistance and permit greater flow between cells ^39^. Thus, one may speculate that the decrease in ZO-1α-minus protein may indicate an attempt by the RPE to reduce permeability in response to hypoxic stress. In addition, we found *TJP1* transcript levels were decreased in our RNA-seq analysis for all cybrids cultured in hypoxia compared to room-air cultures. Interestingly, we observed no significant change in either the ZO-1α-plus or occludin for all groups of cybrids cultured in hypoxia compared to cultures in room-air. Other work in RPE cells has shown that 24 h of hypoxia can reduce occludin in RPE cells^55,56^. One explanation for our findings is that occludin protein levels may decrease more gradually than transcript levels. While our protein data suggests that 48 h of hypoxia does not significantly change occludin protein levels in our cybrids, our RNA-seq data shows that *OCLN* transcript levels are decreased for all cybrids cultured in hypoxia, consistent with these previous studies. In both cases, our cybrid populations reacted similarly in response to hypoxic stress. In conclusion, our study suggests that mtDNA haplogroup and diabetic status do not significantly affect tight junction protein levels in response to hypoxia.

## Supplementary Information

Supplementary Information.

## Data Availability

The RNA-seq raw data are available at Gene expression omnibus database (accession number- GSE211611). RNA-seq analysis are available at https://www.ncbi.nlm.nih.gov/geo/query/acc.cgi?acc=GSE211611. All other relevant data are within the article and its supplementary tables.
