# Supplementary material for: Hypoxia-induced transcriptional differences in African and Asian versus European diabetic cybrids
Source: Sci Rep. 2023 Mar 7;13:3818. doi: 10.1038/s41598-023-30518-x (PMC9992459; doi:10.1038/s41598-023-30518-x)

Supplemental Material

Hypoxia-Induced Transcriptional Differences in African and Asian Versus European Diabetic Cybrids

Andrew H. Dolinko[1,2], Marilyn Chwa[2], Kevin Schneider[2], Mithalesh K. Singh[2], Shari Atilano[2], Jie Wu[3], and M. Cristina Kenney*[1,2]

[1] *Department of Pathology and Laboratory Medicine, University of California Irvine, Irvine, California, 92697*

[2] *Department of Ophthalmology, University of California Irvine, Irvine, California, 92697*

[3] *Department of Biological Chemistry, University of California Irvine, Irvine, California, 92697*

Keywords: Diabetic retinopathy (DR); Retinal Pigment Epithelial Cells; African and Asian Mitochondrial DNA Haplogroups; cybrid cell model; RNA-seq

***Corresponding Author:**

M. Cristina Kenney, M.D., Ph.D.
Professor, Director Mitochondrial Research Laboratory
Department of Ophthalmology, Gavin Herbert Eye Institute
University of California Irvine
843 Health Science Road
Hewitt Hall, Room 2028
Irvine, CA 92697-4390
Phone: (949) 824-7603
Email: mkenney@hs.uci.edu

Supplemental Table 1. Euro and [Afr+Asi] cybrids used in these experiments have a similar average age.

	European					African and Asian			
**Cybrid**	**Haplogroup**	**Age**	**Sex**	**DM/ Non-DM**	**Cybrid**	**Haplogroup**	**Age**	**Sex**	**DM/ Non-DM**
11.08	K1c1c	24	M	Non-DM	11.30	L1b1a	54	F	DM
13.103	K1a3	25	F	DM	11.31	L2b2	42	M	Non-DM
13.45	H1e1a	47	F	Non-DM	11.18	D4a6	39	M	Non-DM
16.186	H1	48	M	DM	13.55	D4a2b	48	F	DM
10.06	U5	53	M	Non-DM	11.38	L0a1a1	38	F	Non-DM
16.184	U	60	M	DM	13.124	L1b2a	33	M	DM
14.135	H1c3	75	F	Non-DM	13.126	L1b1a7	40	F	Non-DM
16.190	H	80	F	DM	15.176	L0a1a	32	M	DM
16.185	U5a1a1	68	M	DM					
	Avg Age	53.33		**p = 0.1**		Avg Age	40.75		
	# Male	5				# Male	4		
	# Female	4				# Female	4		
Non-DM	Avg Age	49.75			Non-DM	Avg Age	39.75		
	# Male	2				# Male	2		
	# Female	2				# Female	2		
DM	Avg Age	56.20			DM	Avg Age	41.75		
	# Male	3				# Male	2		
	# Female	2				# Female	2		

Cybrids are listed in pairs of Non-DM and DM cybrids of similar mtDNA haplogroup and age. The average ages of Euro and [Afr+Asi] cybrids are not significantly different from each other.

Supplemental Table 2. Status of diabetic retinopathy in the Euro and Afr+Asi DM cybrids

European DM cybrids					African+Asian DM cybrids				
**Cybrid**	**Haplogroup**	**Age**	**Diabetes Type**	**DR Status**	**Cybrid**	**Haplogroup**	**Age**	**Diabetes**	**DR Status**
13.103	K1a3	25	Type 2	Mild/No DR	11.30	L1b2a	54	Type 2	NPDR
16.182	L	78	Type 2	Severe NPDR	13.55	D4a2b	45	Type 2	Background DR
16.184	U	60	Type 2	NPDR	13.124	L1b2a	31	Type 1	No DR
16.185	U5a1a1	68	Type 2	NPDR	15.176	L0a1a	32	Type 2	No DR
16.186	H1	48	Type 2	NPDR					
16.190	H	80	Type 2	Background DR					

NPDR- Non-proliferative diabetic retinopathy; DR- Diabetic retinopathy

Supplementary Table 3. Antibodies Used In These Experiments.

Antigen	Antibody	Primary/Secondary	Dilution	Protein Band Size(s)
ZO-1 (TJP1)	ZO-1 Polyclonal Antibody (Cat #40-2200; ThermoFisher Scientific)	Primary	1:1,000	225 kDa (ZO-1 plus) 200 kDa (ZO-1 minus)
Occludin (OCLN)	Occludin Polyclonal Antibody (Cat #71-1500; ThermoFisher Scientific)	Primary	1:1,000	29 kDa (isoform)
Beta-Actin	β-actin (13E5) Rabbit mAb (Cat# 4970; Cell Signaling Technology, Danvers, MA)	Primary (Housekeeping Protein)	1:10,000	45 kDa
Rabbit IgG	Anti-Rabbit IgG, HRP-linked Antibody (Cat# 7074; Cell Signaling Technology)	Secondary	1:10,000	N/A

Supplemental Table 4. Transcripts of endocytosis-associated genes show similar changes between room air and hypoxia for all cybrids.

tracking_id	11-31 normoxia	11-31 HYPOXIA	11-30 normoxia	11-30 HYPOXIA	11.38 normox	11.38 HYPOX	13.124 normox	13.124 HYPOX
CLTA	208.019	249	156.142	294.979	186.529	226.353	149.928	220.616
MERTK	0.837731	0.414042	1.22734	0.253667	1.08911	0.762945	2.39124	0.93357
RAC1	152.373	144.824	146.348	145.885	167.052	153.58	143.093	151.286

13.45 H normox	13.45 H HYPOX	16.186 normox	16.186 HYPOX	11.08 normox	11.08 HYPOX	13.103 normox	13.103 HYPOX
248.192	259.53	155.08	283.733	159.779	202.7	153.008	247.348
0.931135	0.38165	0.498627	0.147659	1.64637	0.832872	3.02166	0.756854
149.003	145.005	129.143	165.542	153.968	186.161	130.755	145.597

14-135 normoxia	14-135 HYPOXIA	16-190 normoxia	16-190 HYPOXIA	11-18 normoxia	11-18 HYPOXIA	13-55 normoxia	13-55 HYPOXIA
287.558	270.225	152.73	226.074	158.675	255.759	221.554	251.753
0.326303	0.102863	0.292812	0.114299	1.43579	0.737189	0.953007	0.560644
137.126	173.049	164.228	170.756	141.414	151.667	180.602	169.248

13-126 normoxia	13-126 HYPOXIA	15-176 normoxia	15-176 HYPOXIA	10-06 normoxia	10-06 HYPOXIA	16-184 normoxia	16-184 HYPOXIA
128.969	159.457	138.795	214.157	108.102	143.012	98.1176	157.432
3.2417	0.902009	0.649644	0.291492	2.21566	1.5334	0.528258	0.295764
145.21	179.387	112.474	132.262	154.862	183.681	112.683	166.004

Values are expressed as Reads-per-Kilobase-per-Million matched reads (RPKM).

Supplemental Table 5. Transcripts of tight junction protein genes are lower for all cybrids cultured in hypoxia than in room air.

tracking_id	11-31 normoxia	11-31 HYPOXIA	11-30 normoxia	11-30 HYPOXIA	11.38 normox	11.38 HYPOX	13.124 normox	13.124 HYPOX
OCLN	1.0998	1.47645	2.21595	0.650693	2.24395	1.39374	2.49914	2.01838
TJP1	3.39907	2.76345	4.96728	1.96341	4.90931	2.85099	5.74854	3.53912

13.45 H normox	13.45 H HYPOX	16.186 normox	16.186 HYPOX	11.08 normox	11.08 HYPOX	13.103 normox	13.103 HYPOX
0.976347	1.15972	2.29511	1.00747	3.02433	2.72341	2.06283	0.856506
2.4331	2.61054	3.07859	0.999963	6.54895	3.08761	4.47968	2.17855

14-135 normoxia	14-135 HYPOXIA	16-190 normoxia	16-190 HYPOXIA	11-18 normoxia	11-18 HYPOXIA	13-55 normoxia	13-55 HYPOXIA
0.992384	1.20245	2.94434	2.59705	1.94416	1.55445	1.14019	1.40275
1.22128	1.10268	9.6376	2.62006	3.84719	2.46155	2.97211	2.76858

13-126 normoxia	13-126 HYPOXIA	15-176 normoxia	15-176 HYPOXIA	10-06 normoxia	10-06 HYPOXIA	16-184 normoxia	16-184 HYPOXIA
4.27803	2.67753	1.93878	1.46684	5.22227	2.72177	3.28947	2.96846
6.78457	3.75345	3.22741	1.41463	6.56698	5.03422	3.77525	3.87615

Values are expressed as Reads-per-Kilobase-per-Million matched reads (RPKM).

Supplementary Figure 1. Cybrids Used in These Experiments Have Similar mtDNA Copy Numbers. DNA from cybrids grown in room air was measured by qRT-PCR against the mitochondrial gene *MT-ND2* and the nuclear gene *18S*, and the ratio *MT-ND2* / *18S* was calculated for each cybrid. Within each mtDNA background, values were normalized to those of the non-diabetic cybrids. (n = 7 Euro/Non-DM, n = 4 Euro/DM, n = 4 Afr+Asi/Non-DM, n = 4 Afr+Asi/DM; 4 independent experiments total) ns, * = $p < 0.05$, ** = $p \le 0.01$, *** = $p \le 0.001$

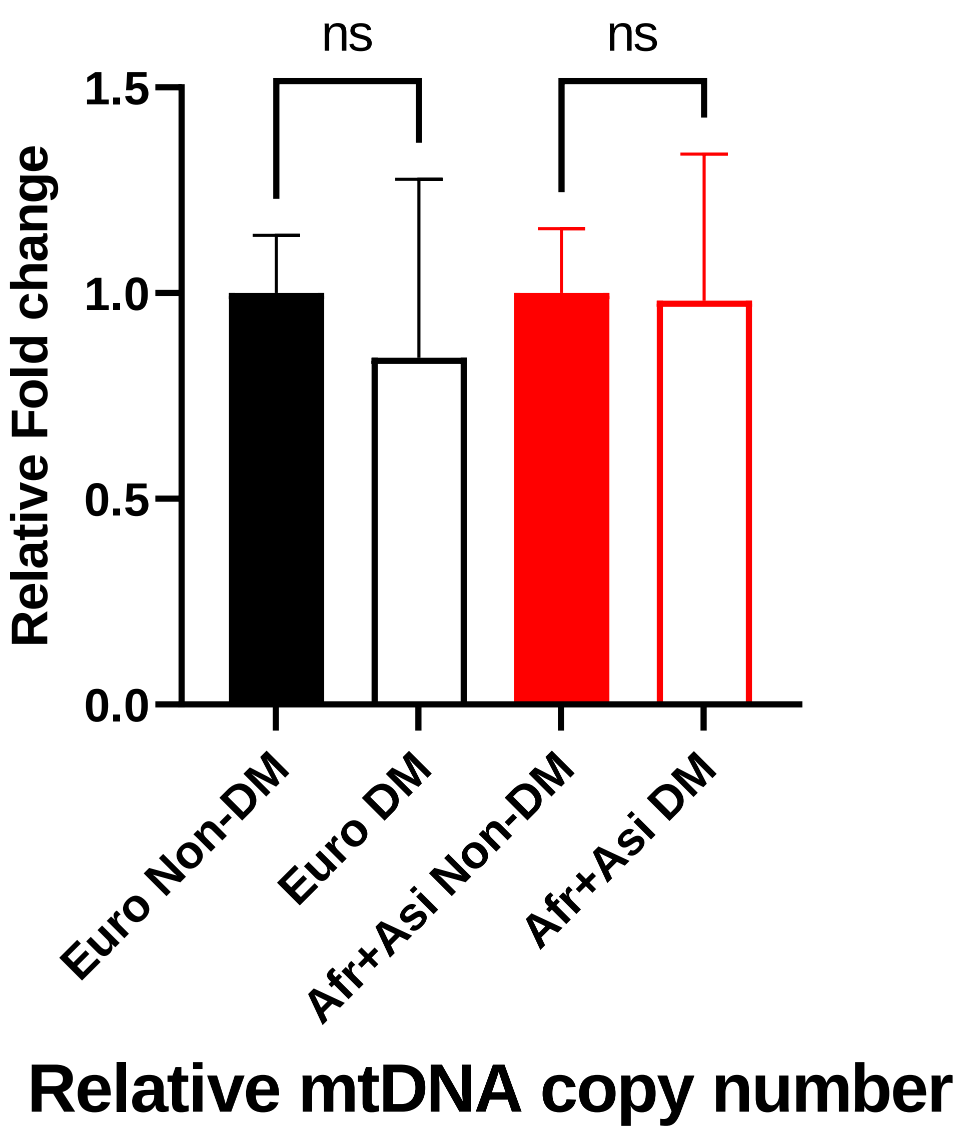

Complete blots

<u>Detail concerning Western blot analysis:-</u>

We ran 8 sets of blots for these experiments. Blots #1,3, 5, and 7 were run and then cut into 2 pieces so that the Upper Part could be exposed to antibodies to ZO-1Plus (225 kDa) and the Lower Part could be exposed to the Occludin antibody (29 kDa). Blots #2, 4, 6, and 8 were also run as intact blots and then cut above the 75kDa marker. The Upper Part was exposed to the ZO-1 Minus antibody (200 kDa), while the Lower Part was exposed to the antibody for β-actin (42 kDa).

For more detail: Briefly, we separated the proteins using a 4-12% gradient gel. We then transferred these proteins from the gradient gels to Blot #1 -8 membrane. After wrapping in plastic wrap, the bright field image was obtained without the ECL camera (Panel a, Bright filed image). After blocking the membranes, we cut the membranes above the 75kDa ladder so it contained proteins larger than 75kDa (Upper Part). The Blots #1, 3 and 5 Upper Parts were exposed to antibodies for ZO-1 and the band for ZO-1 Plus (225 kDa) was measured. Blots #1, 3, 5and 7 Lower Parts were exposed to antibodies to Occludin (29 kDa). The Blots #2, 4, 6, and 8 Upper Parts were exposed to antibodies to ZO-1 antibodies and the band for ZO-1 Minus was measured (200 kDa). The Blots #2, 4, 6, and 8 Lower Parts were exposed to the beta actin antibody (42 kDa). The images for the Upper Part and Lower Part blots were captured using the ECL camera on the Biorad Gel Doc (BioRad, Hercules, CA). The Biorad Gel Doc allows us to take images with either high exposure (Panels b) or low exposure (Panels c) so the variations in intensities were captured. We updated this information in the Materials and Methods section on western blotting in the revised manuscript.

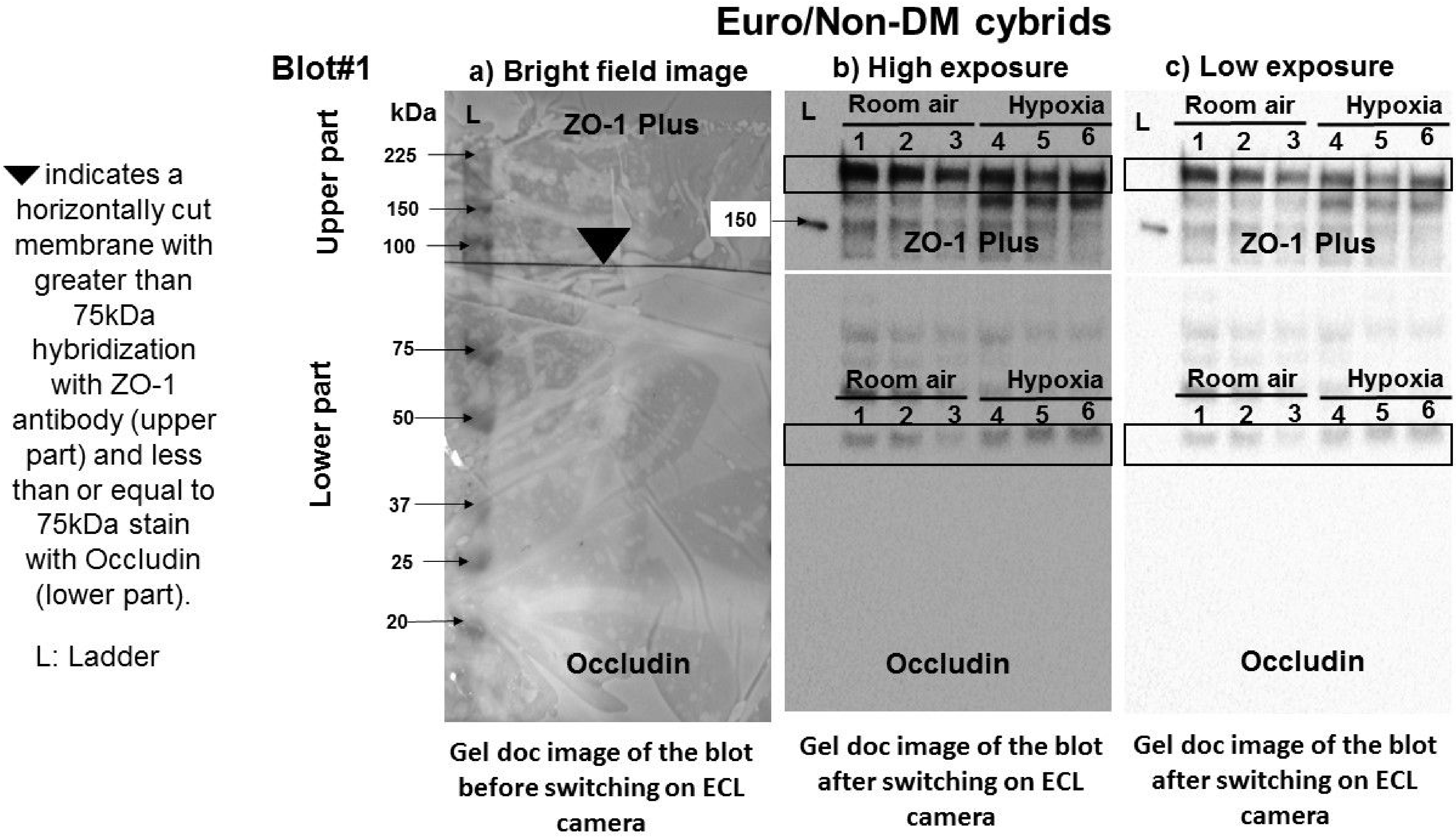
Euro/Non-DM cybrids
Blot#1
a) Bright field image
b) High exposure
c) Low exposure
▼ indicates a horizontally cut membrane with greater than 75kDa hybridization with ZO-1 antibody (upper part) and less than or equal to 75kDa stain with Occludin (lower part).
L: Ladder
Upper part
Lower part
kDa
L
225
150
100
75
50
37
25
20
ZO-1 Plus
Occludin
Room air
Hypoxia
1 2 3 4 5 6
150
Gel doc image of the blot before switching on ECL camera
Gel doc image of the blot after switching on ECL camera
Gel doc image of the blot after switching on ECL camera

Euro/Non-DM cybrids

▼ indicates a horizontally cut membrane with greater than 75kDa hybridization with ZO-1 antibody (upper part) and less than or equal to 75kDa stain with β–actin (lower part).

L: Ladder

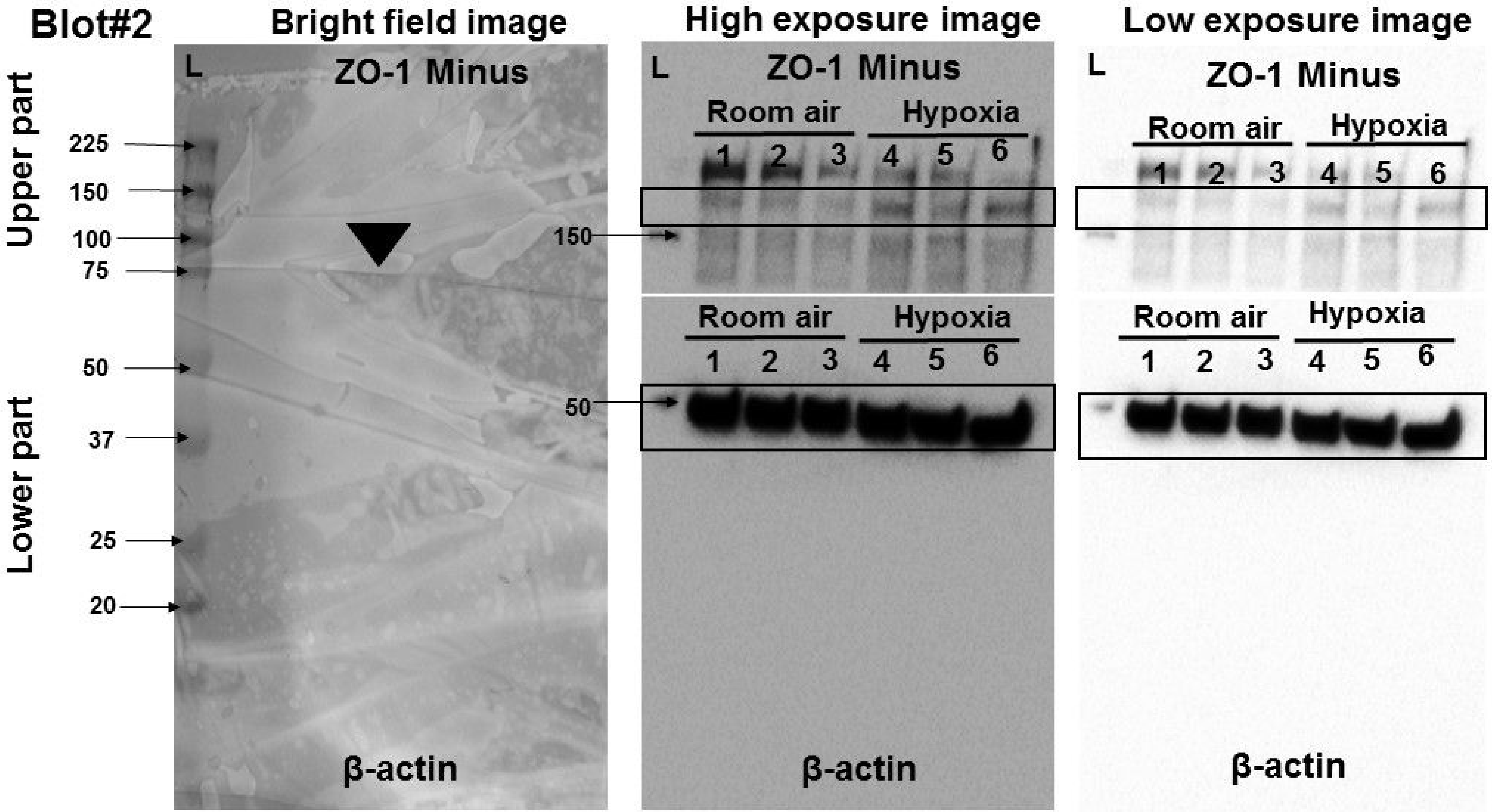

Gel doc image of the blot before switching on ECL camera

Gel doc image of the blot after switching on ECL camera

Gel doc image of the blot after switching on ECL camera

Euro/DM cybrids

▼ indicates a horizontally cut membrane with greater than 75kDa hybridization with ZO-1 antibody (upper part) and less than or equal to 75kDa stain with Occludin (lower part).

L: Ladder

Blot #3

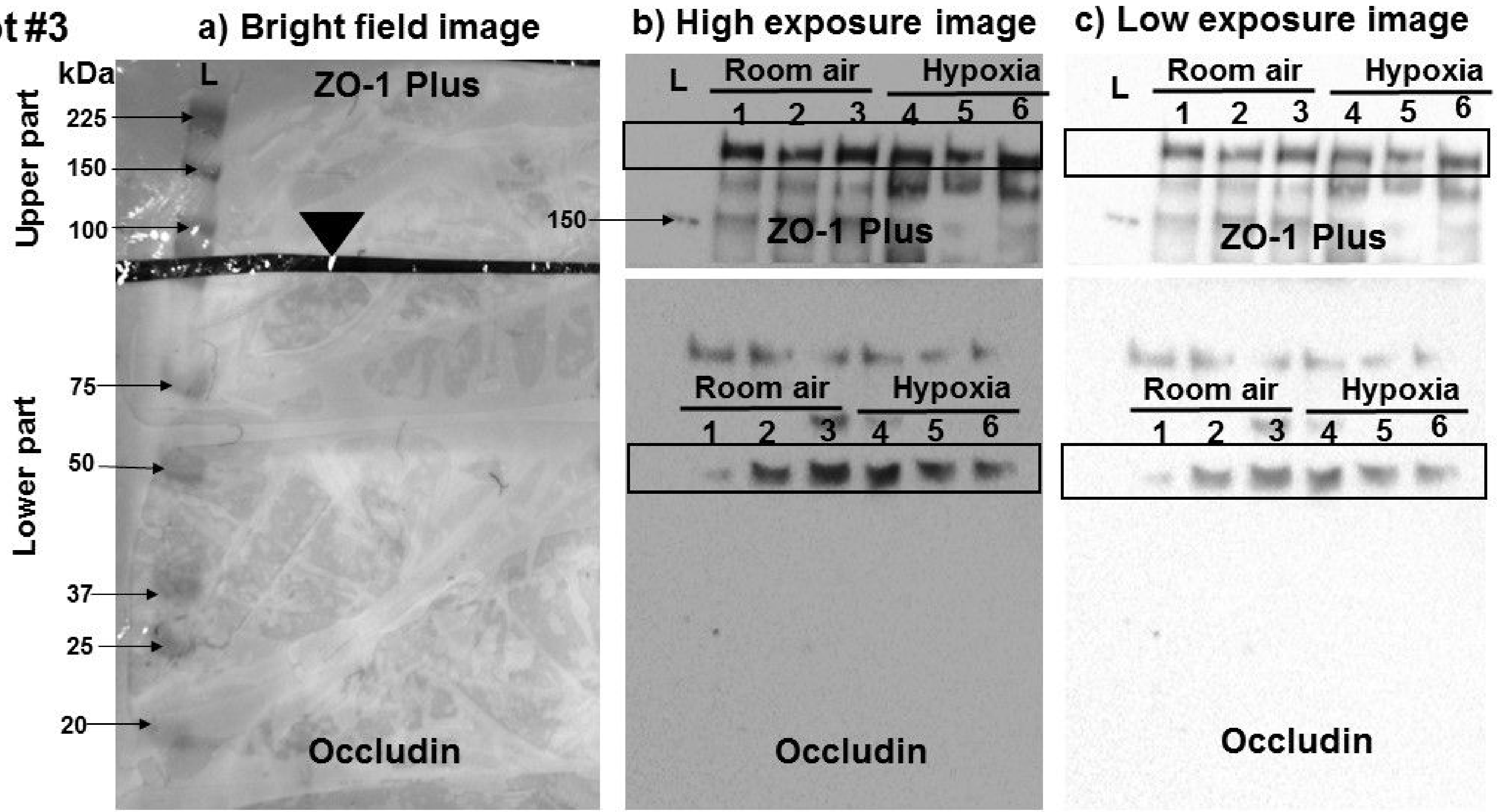

Gel doc image of the blot before switching on ECL camera

Gel doc image of the blot after switching on ECL camera

Gel doc image of the blot after switching on ECL camera

Euro/DM cybrids

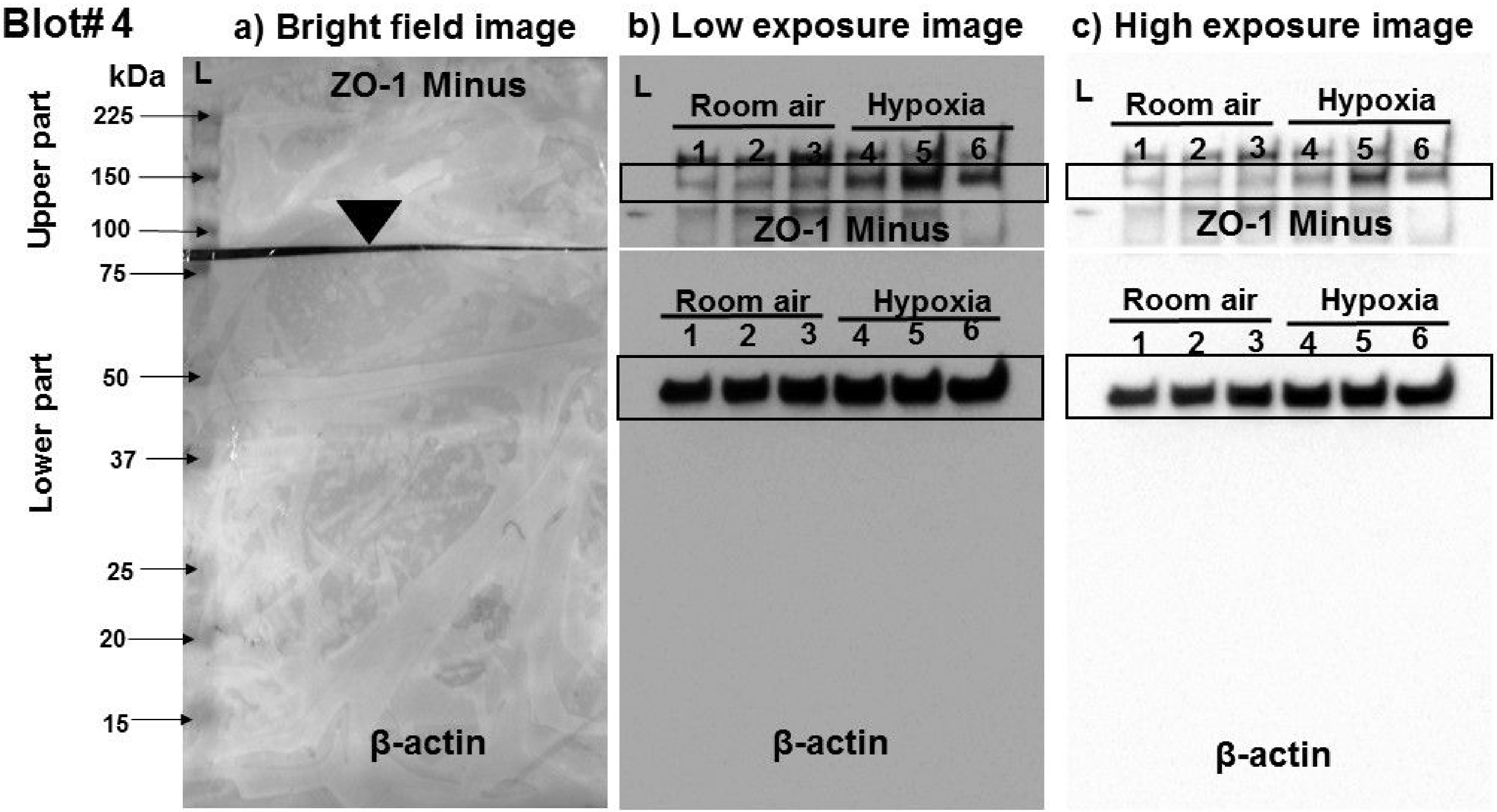

Gel doc image of the blot before switching on ECL camera

Gel doc image of the blot after switching on ECL camera

Gel doc image of the blot after switching on ECL camera

▼ indicates a horizontally cut membrane with greater than 75kDa hybridisation with ZO-1 antibody (upper part) and less than or equal to 75kDa stain with β–actin (lower part).

L: Ladder

Afr+Asi/Non-DM cybrids

Blot #5

▼ indicates a horizontally cut membrane with greater than 75kDa hybridization with ZO-1 antibody (upper part) and less than or equal to 75kDa stain with Occludin (lower part).

L: Ladder

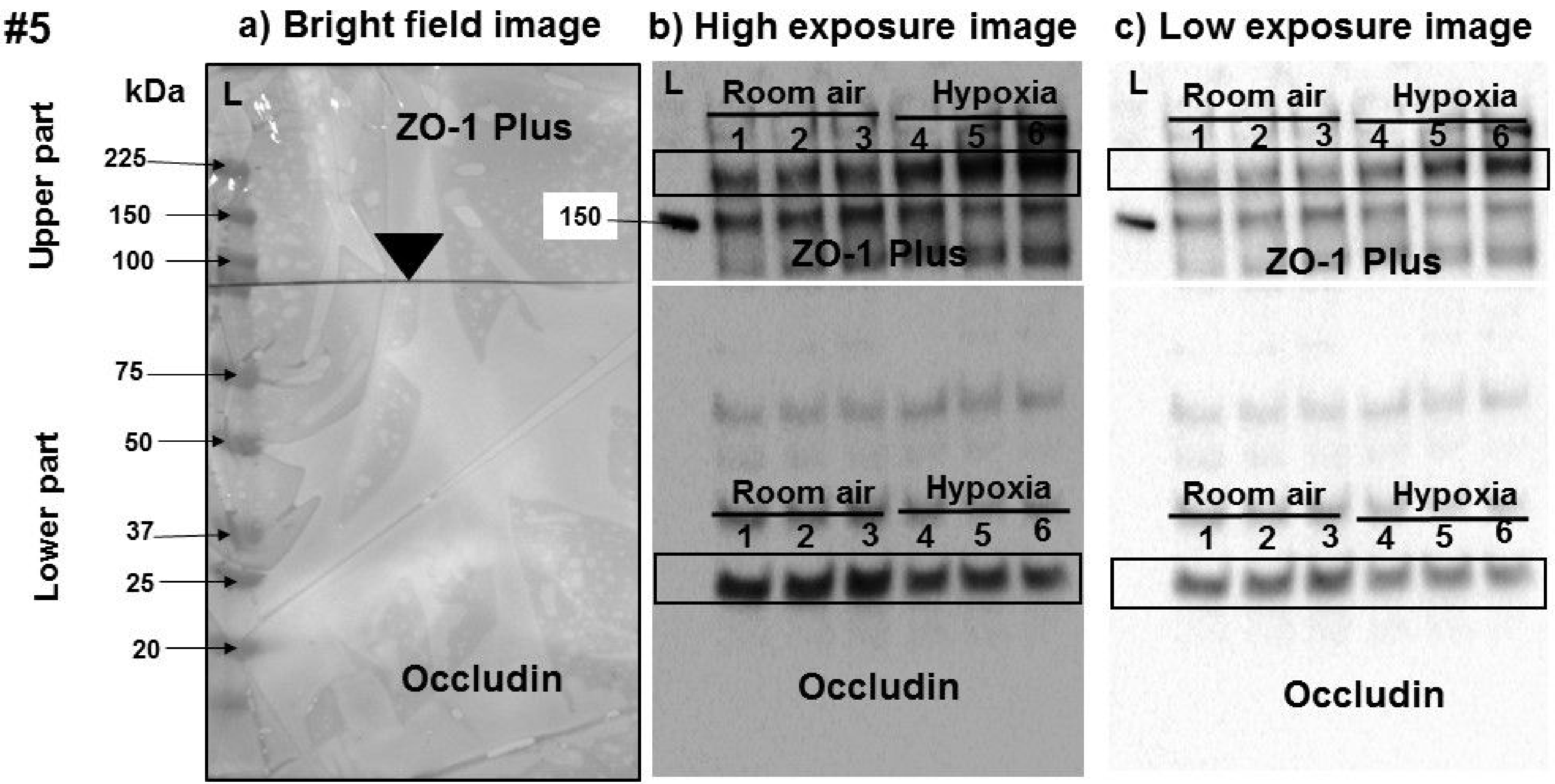

Gel doc image of the blot before switching on ECL camera

Gel doc image of the blot after switching on ECL camera

Gel doc image of the blot after switching on ECL camera

Afr+Asi/Non-DM cybrids

▼ indicates a horizontally cut membrane with greater than 75kDa hybridization with ZO-1 antibody (upper part) and less than or equal to 75kDa stain with β–actin (lower part).

L: Ladder

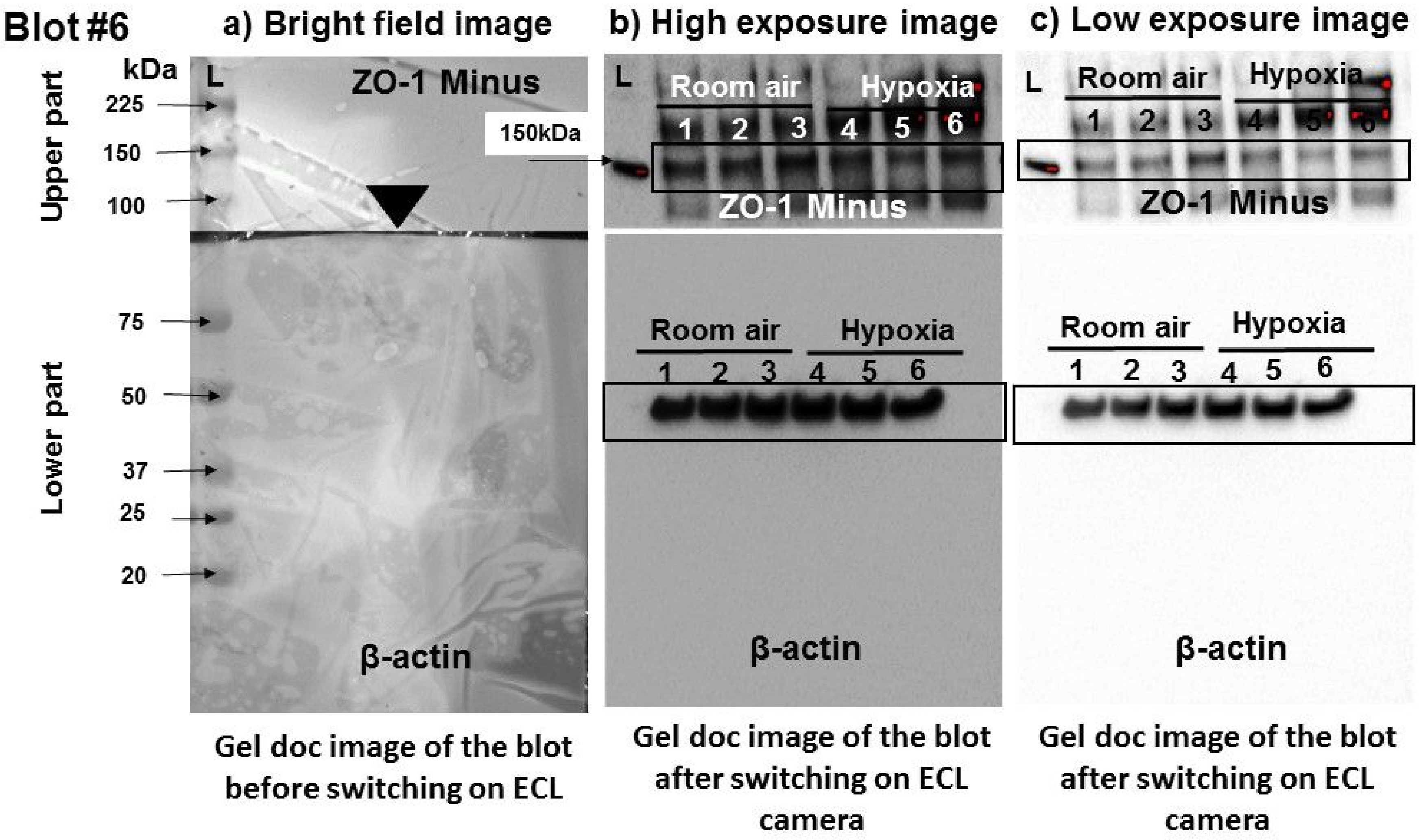

Afr+Asi/DM cybrids

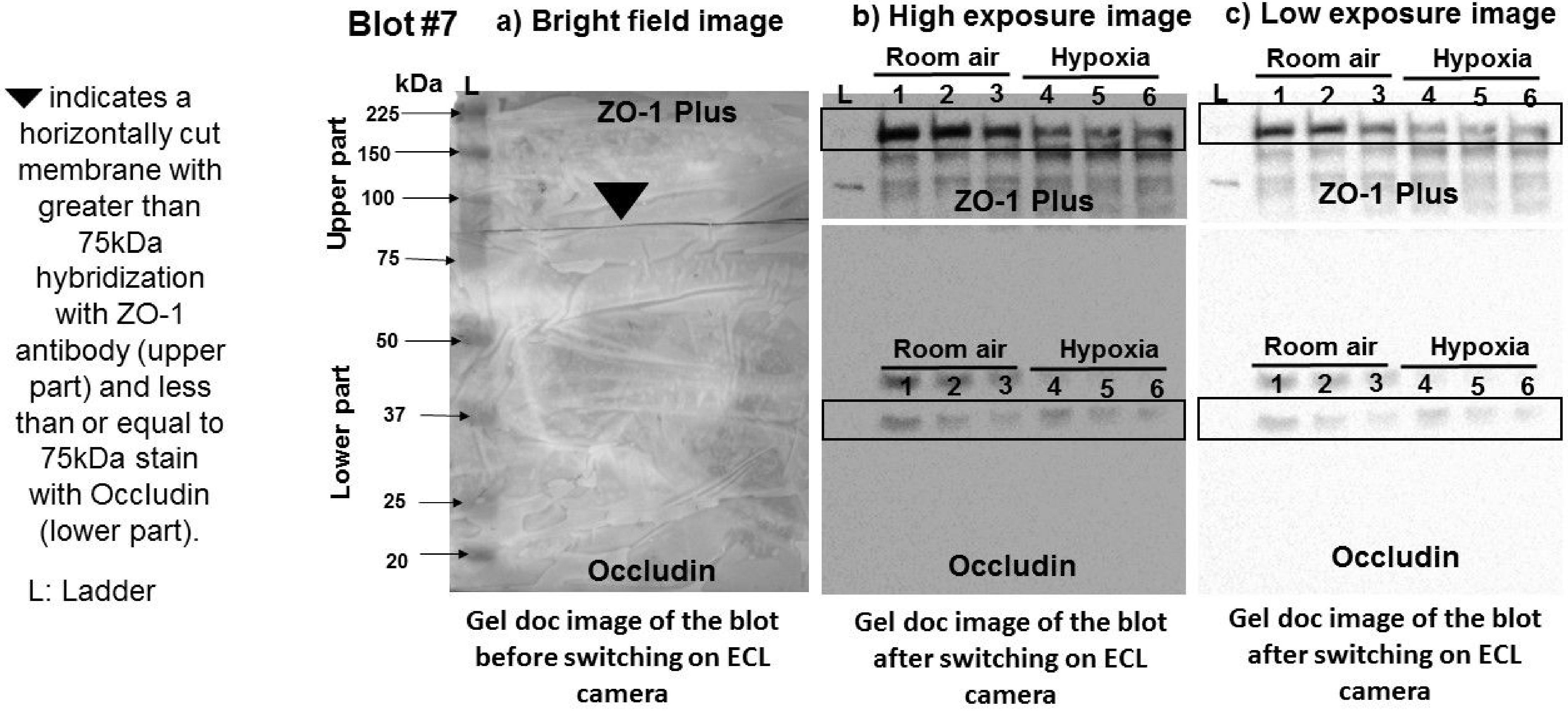

Afr+Asi/DM cybrids

▼ indicates a horizontally cut membrane with greater than 75kDa hybridisation with ZO-1 antibody (upper part) and less than or equal to 75kDa stain with β–actin (lower part).

L: Ladder

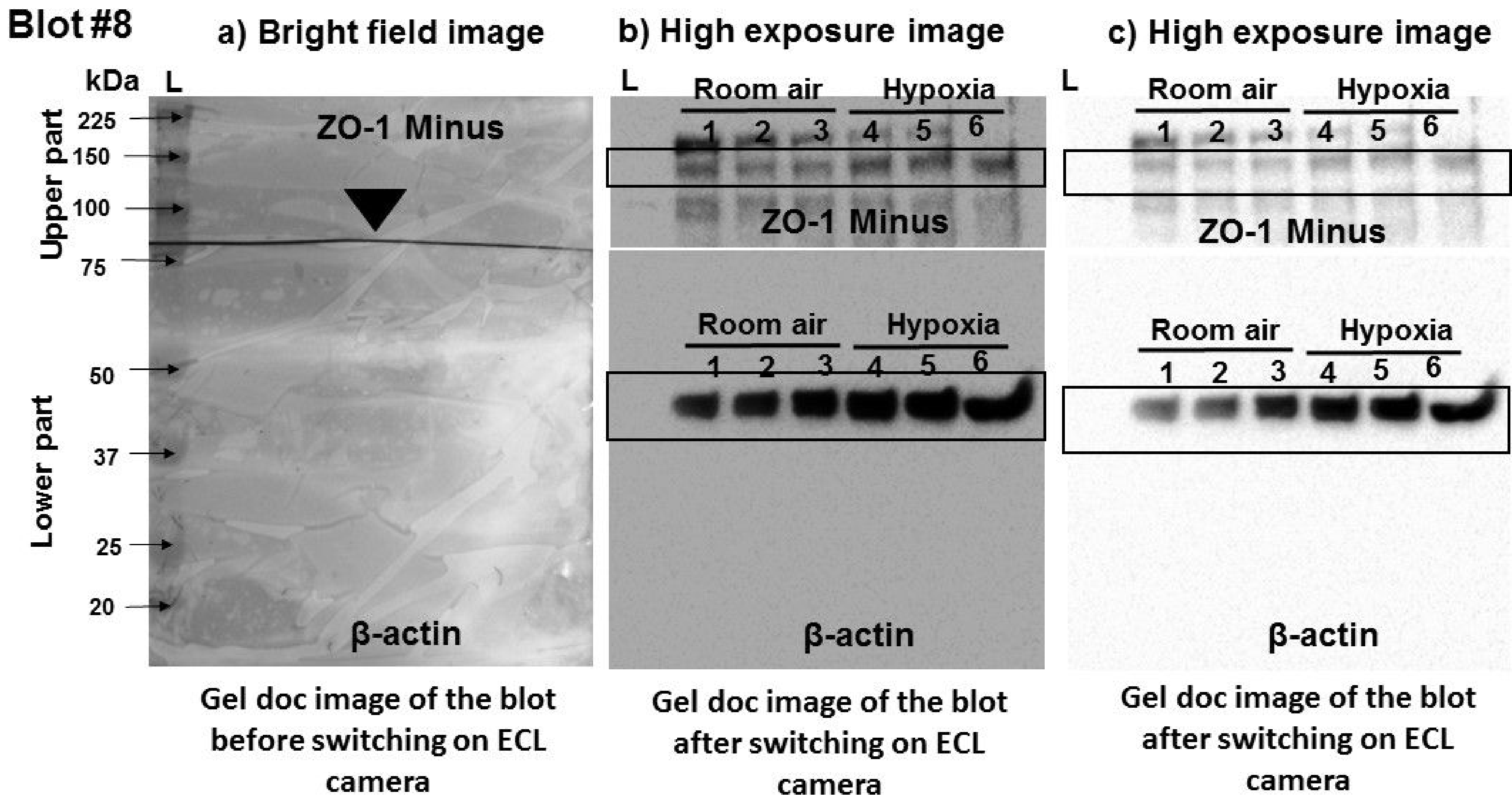

Supplement: Supplementary file 1 — Supplementary Information. [file 41598_2023_30518_MOESM1_ESM.pdf]
